# The effect of exercise on anxiety in the elderly worldwide: a systematic review and meta-analysis

**DOI:** 10.1186/s12955-020-01609-4

**Published:** 2020-11-11

**Authors:** Mohsen Kazeminia, Nader Salari, Aliakbar Vaisi-Raygani, Rostam Jalali, Alireza Abdi, Masoud Mohammadi, Alireza Daneshkhah, Melika Hosseinian-Far, Shamarina Shohaimi

**Affiliations:** 1 Student Research Committee, Kermanshah University of Medical Sciences, Kermanshah, Iran; 2 Department of Biostatistics, School of Health, Kermanshah University of Medical Sciences, Kermanshah, Iran; 3 Department of Nursing, School of Nursing and Midwifery, Kermanshah University of Medical Sciences, Kermanshah, Iran; 4 School of Computing, Electronics and Maths, Coventry University, London, UK; 5 Department of Food Science and Technology, Ferdowsi University of Mashhad (FUM), Mashhad, Iran; 6 Department of Biology, Faculty of Science, University Putra Malaysia, Serdang, Selangor Malaysia

**Keywords:** Sport, Anxiety, Elderly, Meta-analysis

## Abstract

**Background:**

Physical activity and exercise are among the most important, simplest, and cheapest approaches to anxiety treatment, especially for the elderly. Their positive effects on improvement of mental disorders in the elderly have attracted a considerable level of attention. Therefore, the present study was conducted to determine the effect of sport on reducing anxiety in the elderly using meta-analysis.

**Methods:**

In this study, national and international databases of SID, MagIran, IranMedex, IranDoc, Cochrane, Embase, ScienceDirect, Scopus, PubMed, and Web of Science were searched to find studies published electronically from 1999 to 2019. Heterogeneity between the collected studies was determined using the Cochran's test (Q) and I^2^. Due to presence of heterogeneity, the random effects model was used to estimate the standardized mean difference of sport test scores obtained from the measurement of anxiety reduction among the elderly, between the intervention group before and after the test.

**Results:**

In this meta-analysis and systematic review, 19 papers finally met the inclusion criteria. The overall sample size of all collected studies for the meta-analysis was 841 s. Mean anxiety score before and after intervention were 38.7 ± 5.6 33.7 ± 3.4 respectively, denoting a decrease in anxiety score after intervention.

**Conclusion:**

Results of this study indicates that Sport significantly reduces Anxiety in the Elderly. Therefore, a regular exercise program can be considered as a part of the elderly care program.

## Background

Aging is an inevitable process in all human beings [1]. It is a natural developmental stage in which particular physical, psychological, and social changes occur [2]. In other words, aging is referred to spontaneous and progressive irreversible analytical changes in which both physical and mental forces are significantly impaired [3].

According to the World Health Organization (WHO), it is estimated that, by 2020, the population of people over 65 years of age will account for 20% of the world's population, while about 70% of them live in developing countries [4]. In the elderly, all organs of the body undergo a degree of deterioration in their functions; consequently, many chronic diseases occur in the elderly including cardiovascular diseases (e.g. hypertension, coronary artery disease), skeletal diseases (e.g. arthritis, osteoporosis), and mental disorders (such as anxiety and depression) [4–6].

Although anxiety occurs in all age groups, it is a common disorder in the aging period and is more debilitating in the elderly [6, 7]. The elderly are more prone to stress and anxiety as a result of loss or reduction of self-esteem, reduction of activity and stimulation, loss of friends and relatives, loss of physical independence and chronic diseases, changes in daily life or living environment, fear of death and lack of social support [6, 7]. Over 40 million adults in the US suffer from anxiety disorders [6].

Prevalence of anxiety symptoms in the elderly is 15–52% and anxiety disorders occur in 3–15% of adults and are common in the elderly with chronic diseases in particular [8]. Anxiety mostly manifests as physical symptoms such as insomnia, behavioral, sensory, urinary, cardiovascular, and gastrointestinal disorders in the elderly [9, 10].

Important and negative consequences of anxiety include decreased quality of life, disability and greater need for health services, and increased mortality, and therefore, early identification and appropriate treatment would prevent these consequences [7–12].

Physical activity and sport is among the most important, simplest, cheapest, and available therapeutic approaches, especially for the elderly [13]. Sport makes different parts of muscles to interact with each other. In each joint, there is a desired range of motions that are essential for maximum performance. Flexibility is important, not only for physical activity, but also to prevent injury. Inactivity causes the joints to lose their flexibility, as connective tissues become shorter [14]. Atashzadeh et al. [15] argued that exercise training improves daily activities.

There are several preliminary studies on the effect of exercise training on reducing anxiety in the elderly, and there are inconsistencies between their results. One of applications of meta-analysis studies is to respond to these assumptions and resolve such inconsistencies; therefore, the present study was conducted to determine the effect of sport on alleviating the elderly’s anxiety using meta-analysis.

## Methods

### Method used for searching the papers

In this study, the Persian databases of SID, MagIran, IranMedex, and IranDoc and the international databases of Cochrane, Embase, ScienceDirect, Scopus, PubMed, and Web of Science were searched aimed at finding relevant sources from 1999 until December 2019. The lists of references used in all related papers and reports found in above electronic searches were manually reviewed to find other possible sources. The keywords used to search for resources were selected from the Medical Subject Headings (MeSH) database. The Persian keywords were Sport, Anxiety, and The Elderly and the Latin keywords were Exercise, Aerobic Exercise, Exercise Training, Physical Activity, Anxiety, Elderly, and Old.

### Inclusion criteria for selection of the papers

Papers with the following characteristics were selected for the meta-analysis: (1) original research papers, (2) clinical trial studies, (3) full-text availability, and (4) the studies that investigated the relationship between sport and anxiety in the elderly.

### Exclusion criteria for the papers

Selected studies were evaluated more accurately. Review studies or those which their sample had not been selected among the elderly, as well as duplicate studies, and studies conducted using previous data, were excluded from the meta-analysis. Finally, 26 studies entered the third stage, namely quality evaluation.

### Qualitative evaluation of the studies

Quality of the papers were evaluated based on the selected and relevant items present in the CONSORT checklist; the selected items were study design, background and literature review, place and time of study, outcome, inclusion criteria, sample size, and statistical analysis. Articles fulfilling 6 or 7 criteria are considered as high-quality articles; studies having 2 or above, and articles with less than 2 criteria are considered as medium and low-quality articles respectively [16]. In the present study, 19 papers were included in the systematic review and meta-analysis as they were assessed as medium or high-quality articles, and 7 papers were of low quality and were therefore excluded.

### Extracting the data

All final papers entered into the meta-analysis process were prepared for extraction of their data, by a different checklist. The checklist included the following fields: title of the paper, first author's name, year of publication, place of study, sample size of intervention group, mean sample before and after intervention, standard deviation of sample before and after intervention, and type of intervention.

### Statistical-analysis

Given that the context under study was the effect of sport on the elderly anxiety, frequency and percentage were used to combine the results of various research works. The standardized mean difference index was also used in each study. The I^2^ index was used to investigate homogeneity between studies, and due to heterogeneity in the selected research works, the random effects model was used to amalgamate the reported results and to perform the meta-analysis. When the I^2^ index was less than 25%, heterogeneity was considered as low, the value between 25 and 75% was considered as moderate heterogeneity, and the value more than 75% was regarded as high heterogeneity. P-value less than 0.05 was considered as statistically significant. The funnel diagram and Egger’s test were also conducted to evaluate publication bias.

## Results

**Fig. 1:**
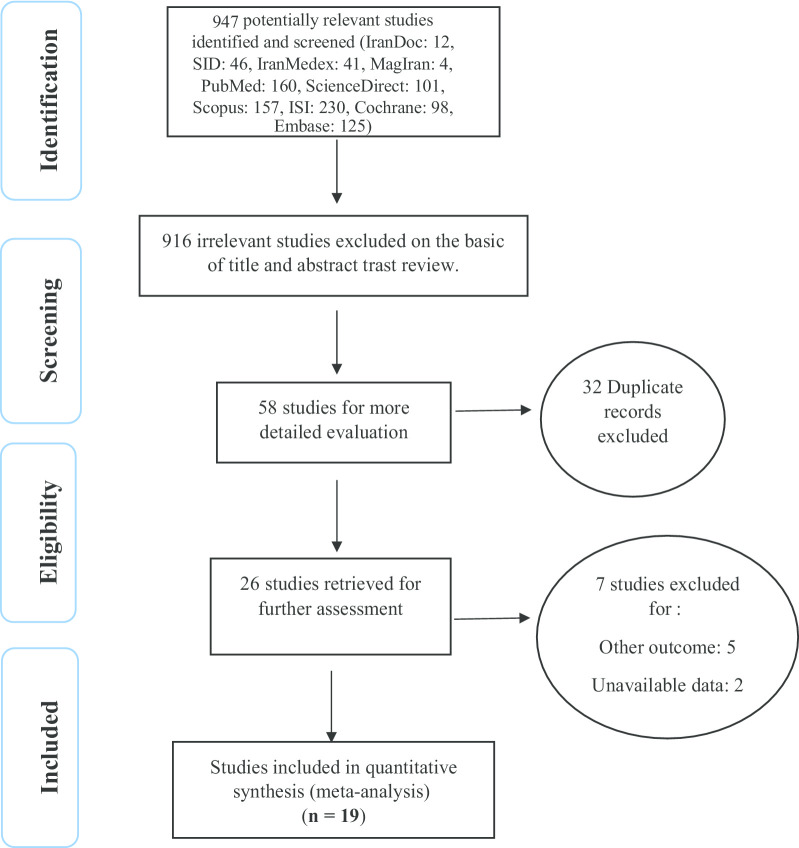
Flow diagram of study selection

In this work, all studies focusing on the effect of sport on the elderly anxiety were systematically reviewed according to the Preferred Reporting Items for Systematic Reviews and Meta-Analyses (PRISMA) guidelines. A total of 974 papers were identified in the initial search, and finally, 19 studies published from 1999 until December 2019 were included in the final evaluation (Fig. 1).

The total number of participants was 841 in the intervention group. Table 1 shows the characteristics of the studies included in systematic review along with the type of administered sport (Table 1).

**Table 1:** Specifications of studies entered into the meta-analysis

Author, year, reference	Place of Type of exercise study	Type of exercise	Sample size	Mean ± SD of before	Mean ± SD of after	Quality
Khesali, 2018, [17]	Iran	Tai Chi Chuan	60	41.79 ± 9.5	29.41 ± 7.64	High
Asiachi, 2017, [18]	Iran	Yoga	30	73.6 ± 6.2	71.3 ± 7.5	Medium
Katula-1, 1999, [19]	USA	Exercise Light	80	14.79 ± 4.85	13.2 ± 4.03	High
Katula-2, 1999, [19]	USA	Exercise Moderate	80	14.13 ± 4.07	13.46 ± 4.0	High
Katula-3, 1999, [19]	USA	Exercise Maximal	80	15.33 ± 5.2	17.16 ± 5.5	High
Cassilhas, 2010, [20]	Brazil	24 weeks of resistance exercise	20	62.91 ± 1.3	56.13 ± 3.81	High
Watanabe-1, 2000, [21]	Japan	Water-based Exercise	36	35.5 ± 7.3	29.1 ± 6.5	Medium
Watanabe-2, 2000, [21]	Japan	Land-based Exercise	37	36.5 ± 7.3	30.5 ± 6.70	Medium
Teixeira-1, 2013, [22]	Portugal	Physical Activity Scale (23)	70	37.24 ± 13.48	34.6 ± 5.71	High
Teixeira-2, 2013, [22]	Portugal	Physical Activity Scale (23)	70	46.68 ± 13.12	36.24 ± 8.82	High
Teixeira-3, 2013, [22]	Portugal	Physical Activity Scale (23)	70	37.29 ± 9.12	37.45 ± 7.57	High
Teixeira-4, 2013, [22]	Portugal	Physical Activity Scale (23)	70	46.48 ± 10.97	38.9 ± 8.15	High
Hyun, 2009, [24]	Korea	Danjeon Breathing Exercise	37	48.69 ± 11.38	41.19 ± 6.88	High
Park, 2011, [25]	Korea	Tai Chi Exercise	23	50.55 ± 8.76	46.91 ± 14.33	High
Bethany-1, 2005, [26]	USA	Yoga	11	35.64 ± 8.82	26.36 ± 8.59	High
Bethany-2, 2005, [26]	USA	Aerobics	11	29.73 ± 8.78	29.0 ± 6.72	High
Bethany-3, 2005, [26]	USA	Walking	10	33.1 ± 8.94	32.5 ± 7.49	High
Antunes-1, 2005, [27]	Brazil	Endurance exercise	23	40.78 ± 9.21	28.56 ± 5.5	Medium
Antunes-2, 2005, [27]	Brazil	Endurance exercise	23	35.08 ± 5.34	29.52 ± 4.85	Medium

According to the available data, indices such as the standardized mean difference and relative risk in the studies were used to determine the final significance of the studies. For research works, in which standard deviation of mean (SD) had been reported, the standardized mean difference index was used in the meta-analysis. Results obtained from the meta-analysis showed heterogeneity between the collected studies, heterogeneity results were reported before intervention (I^2^ = 99.8) and after the intervention (I^2^ = 99.6), thus, the random effects model was used to amalgamate the reported results of the studies and to provide an overall approximation.

**Fig. 2:**
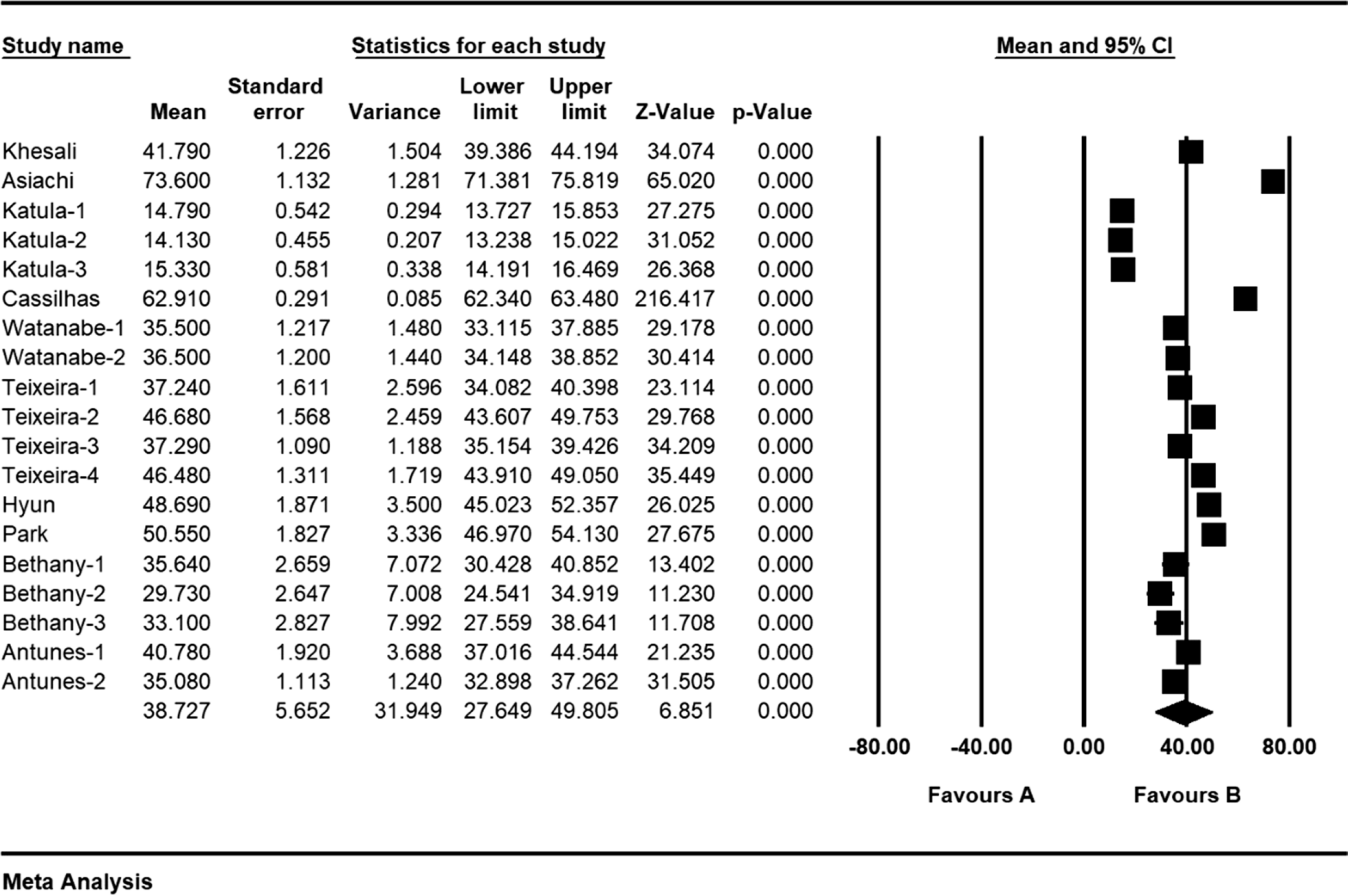
Accumulation diagram of studies included in meta-analysis using standardized mean difference index before intervention

**Fig. 3:**
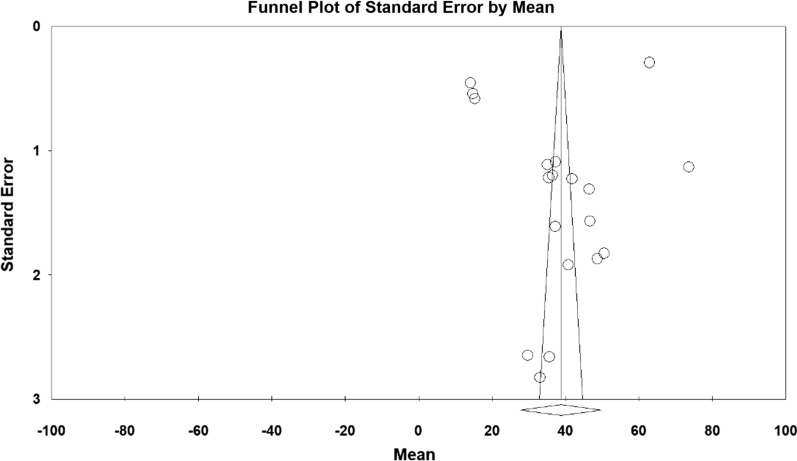
Funnel plot plotted according to the studies included in meta-analysis using standardized mean difference index before intervention

**Fig. 4:**
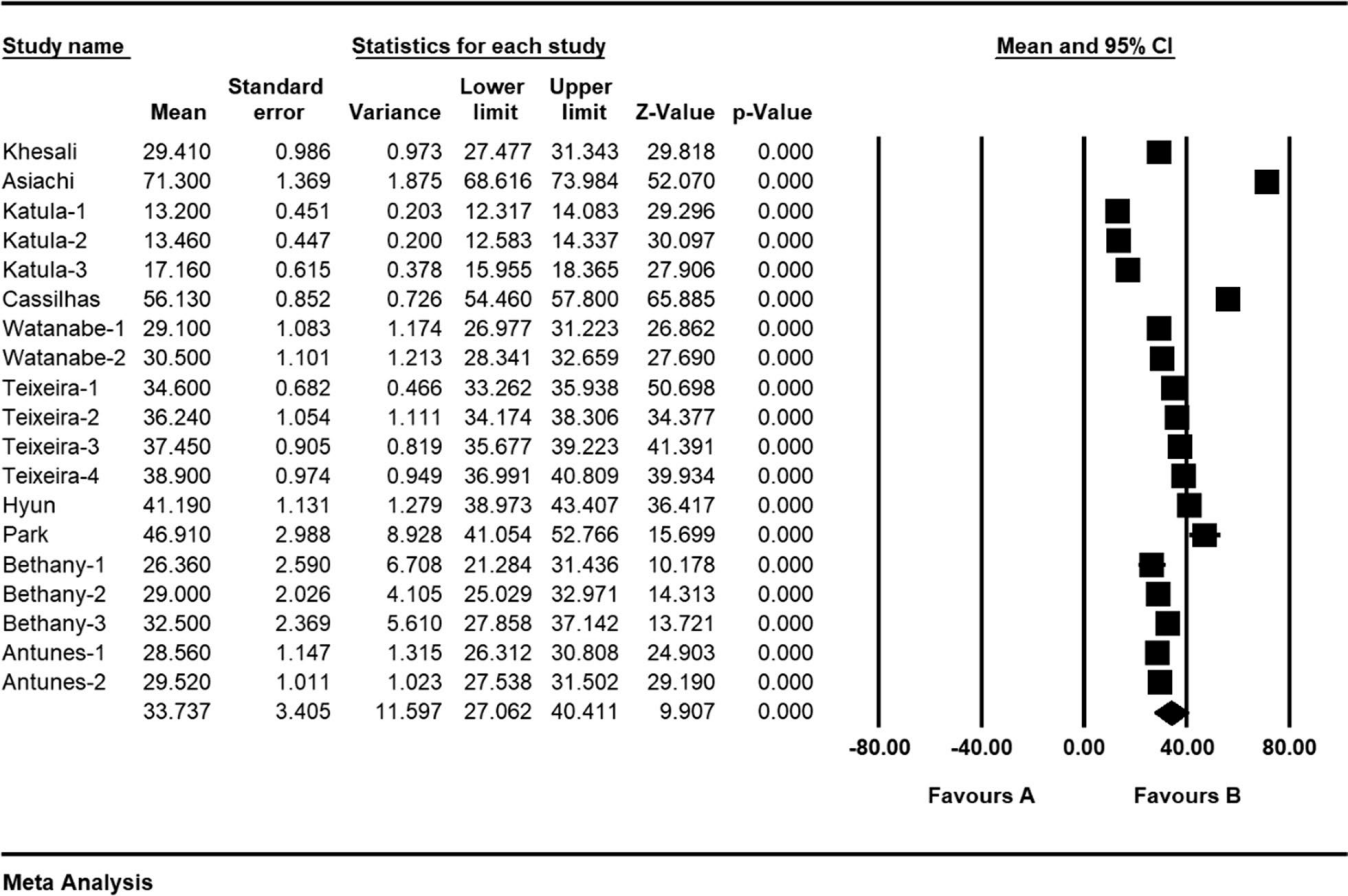
Accumulation diagram of studies included in meta-analysis using standardized mean difference index after intervention

**Fig. 5:**
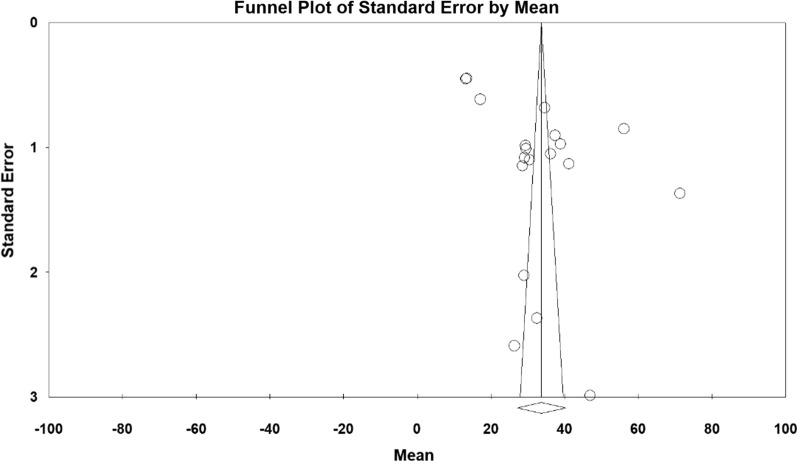
Funnel plot plotted according to the studies included in meta-analysis using standardized mean difference index after intervention

Based on the results obtained from the meta-analysis, the standardized mean difference between the intervention groups was estimated to be 38.7 ± 5.6 and 33.7 ± 3.4 before and after the intervention respectively, indicating that sport reduces anxiety in the elderly. Accumulation graph (Figs. 2 and 3) pesents the standardized mean difference index, as well as the final estimate of the index obtained from combination of all reported results. In this graph, the width of each square also denotes the 95% confidence interval. The Egger's test was used to investigate presence of publication bias in the articles. According to the Egger's test results, there was no pre-intervention (P = 0.549) and post-intervention (P = 0.058) publication bias in the studies (Figs. 4 and 5).

## Discussion

The present study was carried out to determine the effect of sport on reducing anxiety in the elderly using a meta-analysis. Anxiety stems from the inability to solve psychological conflicts, and generally, a large part of the human psychic forces are spent on solving psychological conflicts. For this reason, people with psychological disorders cannot make the best use of their abilities and talents; since mental conflicts deplete their psychic energy and power and cause dissonance in investing for all their psychological dimensions and needs [28].

Results of this study showed that the level of anxiety was high before the test, indicating the effects of anxiety and its consequences on all aspects of life of the elderly.

Therefore, interventions should be made to change the lifestyle and regularly control anxiety of individuals in order to prevent the disorder and reduce its associated complications [28]. Anxiety is primarily preventable and can be controlled and treated in the complication, the elderly need to be thoroughly trained about anxiety and preventing its complications. Besides, the disorder can be controlled and treated through early diagnosis before its complications emerge.

Given the high prevalence of anxiety in the elderly, it is suggested that physicians pay more attention to the symptoms of the disorder and provide appropriate education and training to raise awareness of the individuals, with a view to reduce diagnosis delay.

Results from the present study shows a significant difference in mean scores of pre-test and post-test regarding anxiety severity in the elderly in the intervention group.

Primary benefits of regular exercise, such as increased cardio respiratory fitness, increased muscle strength and endurance, reduced body fatigue, improved morale, and increased ability to perform daily tasks, were found to be greater in the elderly. Moreover, exercise significantly influences and helps to control anxiety and improve general health [29].

Several studies have confirmed positive effects of sport among children, teenagers, adolescents, and adults [17, 18]. Various studies around the world have shown that, sport reduces intensity of anxiety and its recurrence after quitting the sport. Berger and Owen demonstrated that physical exercise has a significant effect on reducing anxiety [31]. DiLorenzo et al. investigated long-term effects of aerobic exercise on anxiety, depression, and emotional states. Their research work showed a relationship between improving participants' physical fitness in aerobic exercises and reducing their anxiety, and anti-anxiety effects of sport continued during a 12-week follow-up after exercise [32].

Anti-Anxiety effects of sport can be explained by a variety of mechanisms, including biological, physiological, and psychological mechanisms of sport. Biologically, sport can have anti-anxiety effects by providing individuals with access to physical fitness; influencing the level of neurotransmitters involved in anxiety, reducing stress hormones, and decreasing muscle tension following exercise activities [33]. Psychologically, sport can reduce anxiety by increasing activity levels, followed by increasing positive conditional reinforcements to the response, providing a situation that distracts individual's attention from threatening stimuli and anxiety, and providing an environment for elevating self-esteem and self-empowerment [33–37]. Experts are advised to use regular sports as a complementary treatment along with medications to help the elderly.

## Limitation

One of the limitations of the present study was completion of the sports survey form by the elderly at their homes as reported in the papers included in this meta-analysis; the survey responses may have been influenced by mental status or inadequate accuracy of the participants’ responses.

## Conclusion

Results of this study showed that sport significantly reduces anxiety in the elderly. Therefore, a regular exercise plan can be considered as a part of the elderly care program.

## Data Availability

Datasets are available through the corresponding author upon reasonable request.

## Abbreviations

WHO: World Health Organization
CONSORT: Consolidated Standards of Reporting Trials
PRISMA: Preferred Reporting Items for Systematic Reviews and Meta-Analysis
